# Sex-specific variations in subgingival microbiome of elderly patients with moderate periodontitis: an exploratory study

**DOI:** 10.3389/fgene.2026.1791446

**Published:** 2026-04-24

**Authors:** Ya-Qiong Zhao, Bi-Fen Kuang, Marie Aimee Dusenge, Qiong Liu, Feng-Yi Zhang, Ying-Hui Zhou

**Affiliations:** 1 Department of Stomatology, Hunan Provincial Engineering Research Center of Digital Oral and Maxillofacial Defect Repair, Hunan Provincial Clinical Research Center for Oral Diseases, The Second Xiangya Hospital, Central South University, Changsha, Hunan, China; 2 College of Medicine and Health Sciences, University of Rwanda, Kigali, Rwanda; 3 Department of Ultrasound Diagnosis, The Second Xiangya Hospital, Central South University, Changsha, Hunan, China; 4 National Clinical Research Center for Metabolic Diseases, Hunan Provincial Key Laboratory of Metabolic Bone Diseases, Department of Metabolism and Endocrinology, The Second Xiangya Hospital of Central South University, Changsha, Hunan, China

**Keywords:** 16S rRNA sequencing, bone loss, elderly, periodontitis, sex-specific variations, subgingival microbiome

## Abstract

**Introduction:**

Periodontitis, a leading cause of alveolar bone destruction and tooth loss, is associated with oral microbiota dysbiosis and shows higher susceptibility in males than in females. This study investigated sex-specific variations in the subgingival microbiome of elderly patients with moderate periodontitis.

**Methods:**

Subgingival plaque samples were collected from 25 patients with moderate periodontitis (8 males, 17 females; aged 50-73 years). The microbial composition was analyzed using 16S rRNA gene sequencing (V3–V4 region). Functional prediction was conducted utilizing the Kyoto Encyclopedia of Genes and Genomes (KEGG) database.

**Results:**

Males exhibited higher Chao1 diversity, and beta diversity analysis revealed sex-based clustering. Wilcoxon rank-sum tests and LEfSe analysis identified *Lactobacillus* was enriched in females. KEGG analysis predicted a trend of enrichment of Immune system and Metabolic pathways in females.

**Conclusion:**

This exploratory study observed sex-specific subgingival microbiome variations of elderly patients with moderate periodontitis. Females exhibited specific enrichment of *Lactobacillus*, which may be associated with predicted Immune system and Metabolic pathways. These findings suggest that sex-specific microbiome differences may be a relevant biological variable in future periodontitis research, and their potential link to alveolar bone loss deserves further exploration.

## 1 Introduction

Periodontitis is one of the most widespread non-communicable diseases globally, affecting 20%–60% of the global population (Wei et al., 2021; Kumar et al., 2022). This chronic inflammatory disease originates from the interaction between a susceptible host and pathogenic biofilm, leading to periodontal structural damage, alveolar bone resorption, and tooth loss (Van der Velden, 2000; Yang et al., 2024). The disease progresses through complex host-microbe interactions modulated by various factors (Van der Velden, 2000; Topcuoglu and Kulekci, 2015), with current diagnosis relying on clinical parameters including attachment loss and probing depth (Hirtz et al., 2021). Multiple microbial biomarkers have been proposed as diagnostic biomarkers of periodontitis, the condition can be successfully controlled with proper treatment, allowing tooth retention for life (Gürsoy et al., 2025; Orishko et al., 2000). Therefore, understanding the relationship between the oral microbiome and host health could improve diagnosis and the targeted treatment of periodontitis and its associated bone loss.

Subgingival plaque microbiota has been extensively documented as key etiological agents in the initiation, pathogenesis, and progression of periodontal diseases (Abusleme et al., 2000). Periodontopathogens including *Porphyromonas gingivalis*, *Aggregatibacte actinomycetemcomitans*, *Tannerella forsythia*, *Peptostreptococcus micros*, *Fusobacterium nucleatum*, *Prevotella intermedia*, and the red complex bacteria (Alghamdi and Almarghlani, 2019). Contemporary research has altered our understanding of the subgingival microbiome ecology through the application of 16S rRNA gene sequencing technology (Abusleme et al., 2000). Advanced molecular techniques now enable detailed characterization of microbial shifts from health to periodontitis through high-throughput sequencing of the V3-V4 region (Abusleme et al., 2000). These culture-independent methods have unveiled significant discrepancies in the composition of microbiome across periodontal health states (Iniesta et al., 2023). Emerging evidence suggests potential demographic variations in periodontal microbiota. Recent studies have indicated possible sex-specific variations in the composition of oral microbiome (Del Pinto et al., 2024). We recently proposed that sex may be a distinguishing factor in the subgingival microbiome composition in initial periodontitis (Zhao et al., 2021). However, the current literature remains limited regarding consistent gender-based patterns in subgingival microbiome profiles. Further research is needed to investigate the potential role of demographic factors in periodontal microbiota composition and disease progression. The oral microbiome undergoes significant changes with aging due to factors such as diet, oral hygiene practices, and immune function (Yue et al., 2025). Elderly individuals have a high prevalence of periodontitis, with the majority presenting with mild to moderate forms of the disease (Jia et al., 2021). Investigating sex-specific microbial patterns at the moderate stage may provide insights into this common disease phase.

To deepen the understanding of the role of sex in periodontal microbiome of periodontitis, this study used 16S rRNA gene sequencing technology to explore sex-specific variations in the subgingival microbiome of elderly patients with moderate periodontitis.

## 2 Materials and Methods

### 2.1 Participants

This exploratory study enrolled 25 participants (8 males and 17 females) aged 50–73 years diagnosed with moderate periodontitis. All participants were recruited consecutively from the Medical Examination Center at The Second Xiangya Hospital of Central South University according to predefined criteria, with no post-recruitment exclusions. Periodontal disease stages were judged in accordance with Tonetti et al. (Tonetti et al., 2018). Specifically, a clinical attachment loss (CAL) of 3–4 mm and a probing depth (PD) of less than 5 mm were diagnosed as moderate periodontitis (Table 1). Inclusion criteria required ≥15 natural teeth with at least one natural tooth in all six sites. Exclusion criteria comprised: oral disease (ulcer, oral lichen planus, oral leukoplakia, pharyngitis, or other pharyngeal disorders), periodontal treatment within the past 6 months, use of immunomodulators or antibiotics within the past 30 days, infection, recent surgery, trauma, pregnancy, smoking (including both current and former smokers), or systemic disease (malignancy, heart failure, musculoskeletal disorders, and autoimmune disease). This study was approved by The Ethics Committee of The Second Xiangya Hospital of Central South University. All participants provided verbal and written consent. The clinical trial registration number is ChiCTR2100046828 (https://www.chictr.org.cn/bin/project/edit?pid=127247).

**TABLE 1:** Characteristics of elderly patients with moderate periodontitis.

Sample name	Age	Clinical attachment loss	Probing depth
Males_01	71	2–4 mm	3–4 mm
Males_02	65	3–4 mm	2–4 mm
Males_03	61	3–4 mm	2–4 mm
Males_04	58	3–4 mm	2–4 mm
Males_05	57	0–3 mm	1–3 mm
Males_06	55	1–3 mm	2–4 mm
Males_07	53	1–4 mm	2–4 mm
Males_08	51	2–4 mm	2–4 mm
Females_01	66	2–4 mm	1–3 mm
Females_02	57	1–3 mm	2–4 mm
Females_03	57	1–3 mm	2–3 mm
Females_04	73	0–3 mm	1–3 mm
Females_05	58	3–4 mm	2–3 mm
Females_06	50	3–4 mm	2–4 mm
Females_07	52	2–4 mm	2–4 mm
Females_08	51	2–3 mm	2–4 mm
Females_09	61	2–4 mm	2–4 mm
Females_10	52	3–4 mm	2–4 mm
Females_11	59	1–3 mm	2–4 mm
Females_12	57	0–3 mm	1–3 mm
Females_13	54	3–4 mm	2–4 mm
Females_14	60	0–3 mm	1–3 mm
Females_15	60	3–4 mm	2–4 mm
Females_16	63	1–4 mm	2–4 mm
Females_17	51	1–3 mm	2–4 mm

### 2.2 Sample collection

Subgingival plaque samples were collected by inserting a sterile Gracey curette into the base of the periodontal pocket from the buccal and lingual sites of four first molars (if first molars are absent, select second molars). Plaque from multiple sites of the same participant were pooled and placed in phosphate-buffered saline. All samples were stored at −80 °C for 1 month before DNA extraction.

### 2.3 DNA extraction and 16S rRNA gene sequencing

Genomic DNA of samples was isolated by utilizing the DNeasy PowerSoil Kit (Qiagen, Germany) and quantified by spectrometer (NanoDrop™ 2000; Thermo Fisher Scientific, United States). The quality of DNA was verified by agarose gel electrophoresis. OE Biotech (Shanghai, China) performed the 16S rRNA sequencing. The V3-V4 region of bacterial 16S rRNA genes was amplified using primers 343F (5′-TACGGRAGGCAGCAG-3′) and 798R (5′-AGG​GTA​TCT​AAT​CCT-3′). PCR amplification was conducted in a 30 μL reaction volume containing 15 μL of 2×Gflex PCR Buffer, 1 μL of each primer (5 pmol/μL), 50 ng of template DNA, and 0.6 μL of Tks Gflex DNA Polymerase (1.25 U/μL). The cycling conditions were: initial denaturation at 94 °C for 5 min; 26 cycles of 94 °C for 30 s, 55 °C for 30 s, and 72 °C for 20 s; with final extension at 72 °C for 5 min. The amplified products were purified by utilizing AMPure XP beads (Beckman Coulter, United States) and quantified with the Qubit dsDNA Assay Kit (Life Technologies, United States). Pooled libraries were sequenced on an Illumina MiSeq platform (Illumina, United States). All samples were processed and sequenced in a single batch to eliminate batch effects.

### 2.4 Bioinformatics and statistical analysis

In total, 1628200 valid tags were obtained after quality control, ranging from 55769 to 68844 per sample, with an average depth of 65128 tags per sample. Clustering of the original FASTQ format sequencing data was performed using the Vsearch software (version 2.4.2) to construct operational taxonomic units (OTUs) at a 97% similarity level. Representative sequences for each OTU were identified with QIIME (version 1.9.1). Taxonomic assignment was performed by aligning representative sequences against the Greengenes database for 16S rRNA gene annotation. Species annotation was conducted using the RDP classifier with a confidence threshold of >0.7. BLAST software was additionally used for species annotation comparison. Alpha (α-) diversity indices were compared using Wilcoxon rank-sum tests. Beta (β-) diversity was assessed through nonmetric multidimensional scaling (NMDS) and principal coordinates analysis (PCoA) based on unweighted UniFrac distances. Wilcoxon rank-sum test was performed to evaluate the relative abundance of predominant bacteria, followed by the Benjamini-Hochberg false discovery rate (FDR) correction for multiple testing. Linear discriminant analysis (LDA) effect size (LEfSe) analysis was performed using PICRUSt1 as previously described (Segata et al., 2011). LDA score >3.0 was used to identify group-specific biomarkers. Functional predictions were generated using PICRUSt1 based on the 16S sequencing data annotated against the Greengenes database, with Kyoto Encyclopedia of Genes and Genomes (KEGG) pathway annotations. *P* < 0.05 was set as the criterion for statistical significance.

## 3 Results

### 3.1 Characteristics of participants and OTU analysis

**FIGURE 1:**
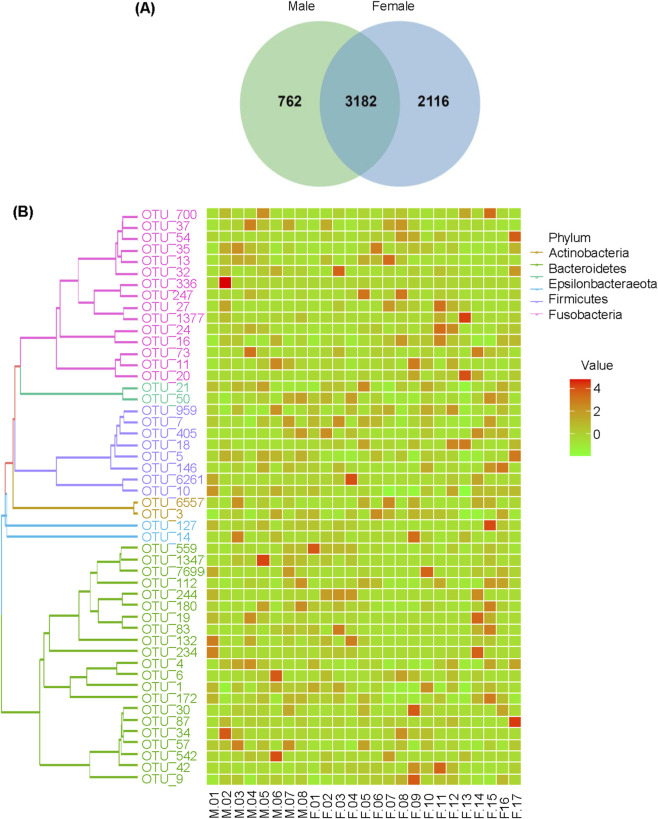
Information of OTUs. **(A)** the Venn diagram. **(B)** The phylogenetic tree (Left) and the abundance map (Right) of the 50 most abundant genera.

A total of 25 participants aged 50–73 years were enrolled in this study, with no significant age difference observed between females and males (*P* > 0.05, Table 2). The subgingival microbiome composition of elderly patients with moderate periodontitis was analyzed using Illumina MiSeq sequencing and QIIME pipeline. The clustering of qualified sequences yielded 6060 OTUs, with 3182 (52.5%) OTUs shared between groups, while 2116 (34.9%) and 762 (12.6%) OTUs were unique to female and male groups respectively. (Figure 1A). The OTU distribution per participant at all levels of classification is depicted in Supplementary Figure S1. A phylogenetic tree was constructed comprising the 50 most abundant genera, with relatively high abundances observed in *Bacteroidetes*, *Proteobacteria*, *Fusobacteria*, *Actinobacteria*, *Firmicutes* and *Epsilonbacteraeota* (Figure 1B).

**TABLE 2:** Age information of the participants.

Group	Number	Age (year)
Males	8	58.875 ± 6.600
Females	17	57.706 ± 6.039
*P*	​	>0.05

**FIGURE 2:**
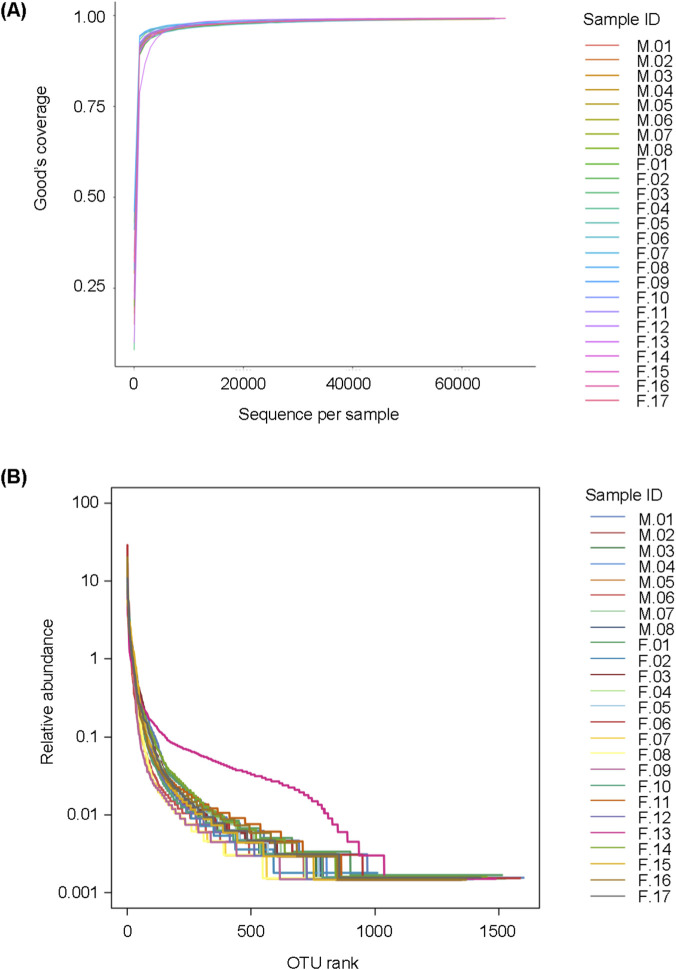
The sequencing quality of samples from elderly male and female patients with moderate periodontitis. **(A)** Rarefaction curves based on Good’s coverage. **(B)** Rank-abundance curve.

Sequencing quality was assessed using rarefaction curves (based on Good’s coverage index) and rank abundance curves. Both curves approached saturation, indicating that the sample number was sufficiently rich to reflect the information of the majority of microbial species. There was no significant difference in the rarefaction curves between the two groups (Figures 2A,B).

### 3.2 Diversity analysis

**FIGURE 3:**
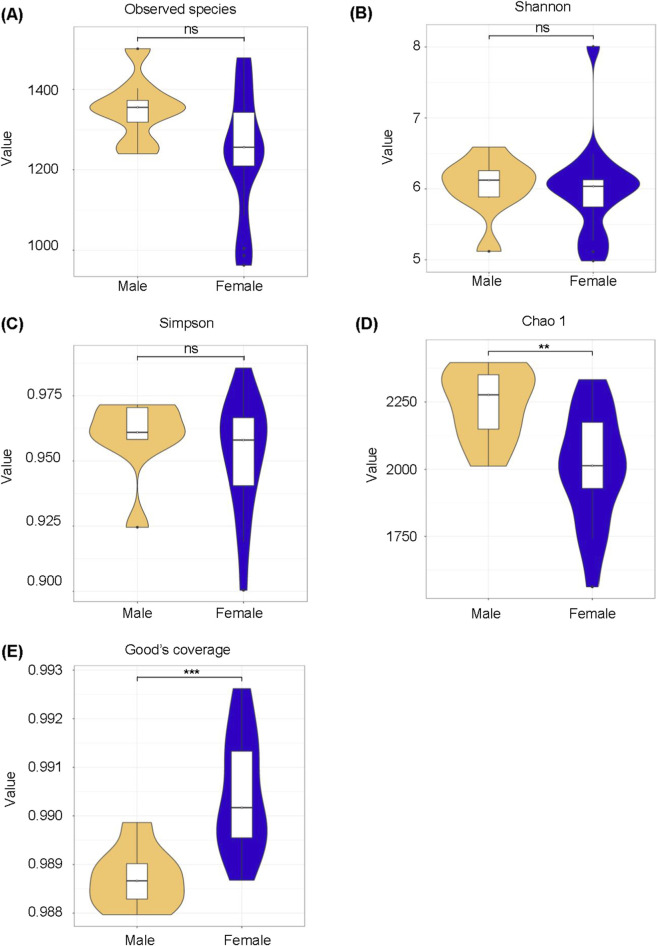
α-diversity analysis of samples from elderly male and female patients with moderate periodontitis. **(A)** Observed species, **(B)** Shannon, **(C)** Simpson, **(D)** Chao 1, and **(E)** Good’s coverage index between males and females. ^∗∗∗^ *P* < 0.001; ^∗∗^ *P* < 0.01; ns: not significant.

The α-diversity reflects the diversity and richness of microbiome. α-diversity indices of Observed species, Shannon, Simpson, Chao1, and Good’s coverage were analyzed at 97% identity to evaluate the diversity and richness of samples. Intergroup statistical comparisons were conducted using the Wilcoxon rank-sum test. Analysis of the Observed species index, Shannon index, and Simpson index did not reveal significant differences. However, the Chao1 index in males was significantly higher than that in females (*P* < 0.01), suggesting greater microbial diversity in males. Although Good’s coverage index showed a statistically significant difference between groups (*P* < 0.001), both groups achieved high coverage values (approximately 0.988), indicating that our sequencing depth successfully captured nearly 99% of the bacterial species present. The statistically significant *P*-value is primarily driven by the extremely low intra-group variance, and the minute numerical difference lacks practical biological significance. Therefore, the sequencing depth is considered adequate for both groups (Figure 3; Table 3; Supplementary Table S1).

**TABLE 3:** α-diversity indices of individual participants.

Samples	Observed species	Shannon	Simpson	Chao1	Goods coverage
M.01	1501	6.588411	0.970259	2233.01	0.988818
M.02	1362.4	5.874005	0.958121	2319.699	0.988109
M.03	1353.5	5.890914	0.958289	2396.564	0.987968
M.04	1335.9	6.045639	0.960294	2121.486	0.989175
M.05	1357.3	6.248913	0.97155	2351.17	0.988348
M.06	1239.9	5.119041	0.924552	2157.108	0.988966
M.07	1403.1	6.278155	0.971165	2351.635	0.988508
M.08	1266.8	6.201425	0.961561	2012.125	0.989864
F.01	1466.7	5.981484	0.94055	2263.368	0.989002
F.02	1004.3	6.127026	0.97122	1561.854	0.99242
F.03	1238.3	6.270015	0.966527	1786.004	0.991051
F.04	1209.5	5.957956	0.956435	2060.381	0.990011
F.05	1270.9	6.048855	0.959072	2173.289	0.989427
F.06	1478.5	5.404525	0.900421	2200.529	0.988675
F.07	986.7	5.113096	0.926256	1763.241	0.991213
F.08	962.4	5.27384	0.945234	1741.24	0.991438
F.09	1085.6	4.983496	0.919144	1972.789	0.990213
F.10	1281.7	5.745736	0.950231	1994.834	0.98972
F.11	1258.1	6.124049	0.957993	1929.382	0.991812
F.12	1220.5	6.036436	0.964299	2074.574	0.989549
F.13	1402.3	8.008821	0.985639	2333.196	0.992618
F.14	1372.7	6.489216	0.971929	2240.954	0.989362
F.15	1212	6.306448	0.975734	2013.768	0.990169
F.16	1256.1	5.798482	0.939299	1947.489	0.991331
F.17	1342.9	6.110389	0.96241	2033.253	0.989691

**FIGURE 4:**
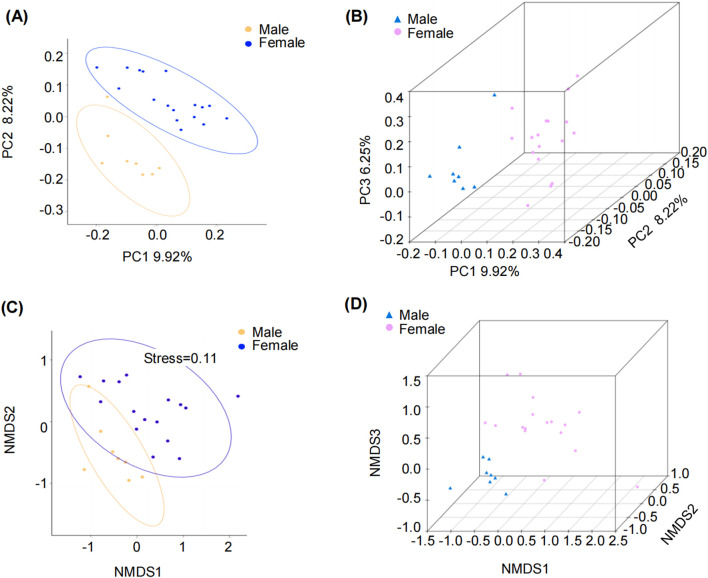
β-Diversity analysis of samples from elderly male and female patients with moderate periodontitis. **(A,B)** principal coordinates analysis (PCoA), and **(C,D)** non-metric multidimensional scaling (NMDS) analysis (two and three-dimensional respectively). Both PCoA and NMDS analyses are based on unweighted UniFrac distances.

β-diversity analysis was performed using PCoA and NMDS based on unweighted UniFrac distances to assess differences in microbial communities between groups. Both PCoA and NMDS analyses revealed spatial separation of microbial communities between male and female groups in two-dimensional and three-dimensional representations. These findings indicate the presence of sex variations in the subgingival microbiome of elderly individuals with moderate periodontitis (Figure 4).

### 3.3 Composition of subgingival microbiome

**FIGURE 5:**
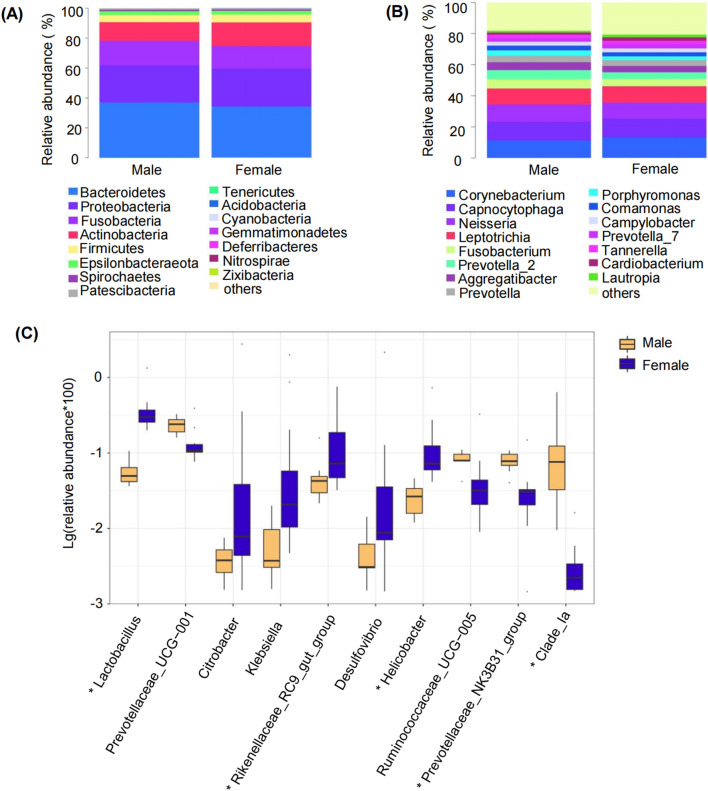
The predominant subgingival plaque microbial composition in elderly male and female patients with moderate periodontitis. **(A)** Phylum level composition. **(B)** Genus level composition. The top 15 taxa are shown, cumulatively representing >99% (phylum) and >80% (genus) of total relative abundance. **(C)** Species difference analysis between males and females by the Wilcoxon rank-sum test at the Genus level. ^*^FDR-adjusted *P* < 0.05.

The relative abundance of the top 15 bacteria at the phylum (Figure 5A) and genus levels (Figure 5B) was assessed. Although the dominant microbial profiles showed similarity between sexes, significant differences were observed in species abundance. To elucidate sex-specific variations in bacterial communities among moderate periodontitis patients, the top 10 differentially abundant species at genus levels were analyzed (Figure 5C). Genus-level analysis revealed *Lactobacillus* as significantly more abundant in female subjects (FDR-adjusted *P* < 0.05).

**FIGURE 6:**
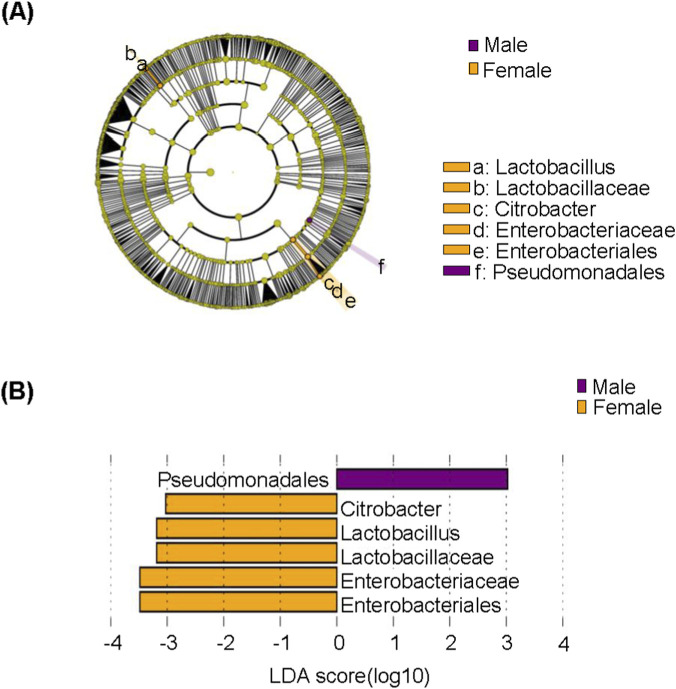
Linear discriminant analysis effect size (LEfSe) analysis of subgingival microbiome in elderly male and female patients with moderate periodontitis. **(A)** Taxonomic cladogram representation of significantly different subgingival microbiome between male and female groups. The colored nodes from the inner to the outer circles represent taxa from the phylum to genus level. The significantly different taxa are indicated by group-specific colors. **(B)**Histogram of linear discriminant analysis (LDA) scores for differentially abundant subgingival microbiome between groups.

LEfSe analysis was performed to recognize subgingival microbiome with abundance differences between elderly males and females with moderate periodontitis. Potential biomarkers were characterized across taxonomic levels (phylum to genus) using LEfSe, and a cladogram was generated to visualize the most discriminative bacterial clades (LDA score ≥3.0). Microbial community diversity analysis revealed significant gender-specific variations in bacterial abundance. Figure 6 showed that *Pseudomonadales* order was markedly increased in males, while female subjects showed significantly elevated levels of *Lactobacillus* genus*,* Lactobacillaceae family, *Citrobacter* genus*,* Enterobacteriaceae family, and *Enterobacteriales* order.

### 3.4 Function prediction

**FIGURE 7:**
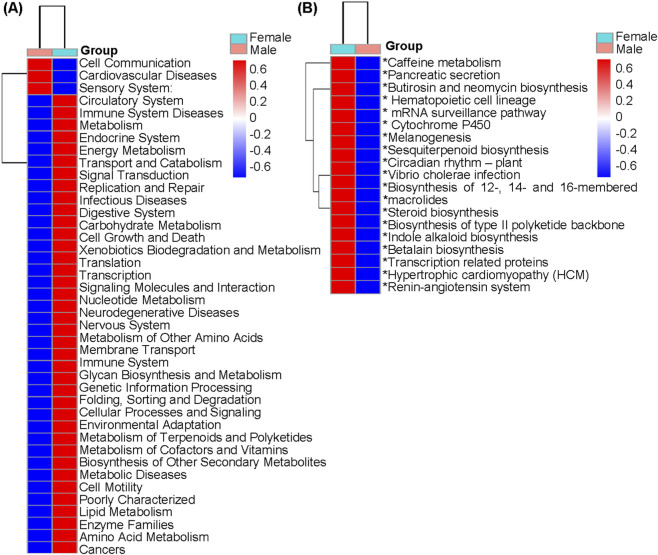
KEGG pathway enrichment analysis. Heat map of predicted pathways between elderly male and female patients with moderate periodontitis. **(A)** Level 2 pathways. **(B)** Level 3 pathways. ^*^ *P* < 0.05.

Comparative analysis of KEGG pathways revealed that PICRUSt predicted sex-specific functional differences in the subgingival microbiome of moderate periodontitis patients. At level 2, male patients showed a trend of predicted upregulation in Cardiovascular diseases, Sensory system, and Cell communication pathways. In contrast, female patients exhibited a trend of predicted enrichment in Immune system pathways and Metabolic pathways (including Amino acid metabolism, Carbohydrate metabolism, and Lipid metabolism) (Figure 7A). Further analysis at level 3 revealed that the Hematopoietic cell lineage pathway, a component of the Immune system pathway at level 2, was predicted significantly upregulated in females (Figure 7B).

## 4 Discussion

This exploratory study investigated sex-specific subgingival microbiome differences in elderly patients with moderate periodontitis using 16S rRNA gene sequencing. The results observed significant compositional variations between males and females. α-diversity analysis revealed higher microbial diversity in males compared to females, while β-diversity showed clear sex-based clustering of samples. The Wilcoxon rank-sum test and LEfSe analysis revealed that *Lactobacillus* was enriched in females. KEGG pathway analysis suggested that sex-specific microbiome differences might be associated with predicted Immune system pathways and Metabolic pathways.

Studies have demonstrated sex-based differences in periodontitis prevalence, with males showing higher susceptibility than females (Del Pinto et al., 2024). The 16S rRNA sequencing approach is particularly valuable for investigating these sex differences as it is a robust method for oral microbiome analysis (Arredondo et al., 2023). Our study employed 16S rRNA gene sequencing to compare subgingival microbiome abundance between male and female patients with moderate periodontitis in the elderly. The Venn diagram analysis revealed that 52.5% of OTUs were shared between sexes in moderate periodontitis patients. Among the two groups, the most abundant phyla were *Bacteroidetes*, *Proteobacteria*, *Actinobacteria*, *Fusobacteria*, *Firmicutes*, and *Epsilonbacteraeota*. These findings align with Salem et al.'s report identifying *Bacteroidetes* and *Firmicutes* as dominant subgingival bacteria in Stage II generalized periodontitis (Abu Fanas et al., 2021), and are consistent with Jiang et al.'s observation of *Firmicutes*, *Bacteroidetes*, *Proteobacteria*, *Fusobacteria*, *Actinobacteria*, and *Saccharibacteria* as major phyla in elderly populations with dental caries (Jiang et al., 2018), suggesting relative stability in major human oral microbiome composition.

Emerging evidence indicates that periodontitis is driven by a mutually reinforcing interaction between a dysregulated microbiome and dysregulated inflammation (Hajishengallis et al., 2000). Our data reveal sex-specific variations in microbiome ecology, with males exhibiting greater subgingival microbiome diversity than female elderly patients with moderate periodontitis. This microbial diversity dimorphism was consistently observed in our prior studies, where males displayed significantly higher phylogenetic diversity (PD whole-tree) and Chao1 indices than females in youths with severe periodontitis (Z et al., 2021). Sex differences in the oral microbiome may be associated with both sex hormones and sex chromosomes. Sex hormones (e.g., estrogen, progesterone, and testosterone) not only modulate host immune responses but also directly influence the composition and function of oral microbial communities (Cornejo Ulloa et al., 2023; Jaillon et al., 2019). Additionally, several genes encoding innate immune molecules are located on the X chromosome (Jaillon et al., 2019), which may be associated with sex-specific differences in the oral microbiome. The observed sex-specific variations in oral microbiota may be associated with more severe periodontal structural damage, alveolar bone resorption, and tooth loss in males. Notably, males exhibited higher Chao1 richness but no difference in Shannon or Simpson indices, suggesting that the increased microbial diversity in males may be driven by rare taxa (DeClercq et al., 2021). This pattern suggests the importance of further investigating the functional roles of rare taxa in understanding sex-specific differences in periodontitis pathogenesis.

LEfSe analysis revealed that males exhibited higher abundance of *Pseudomonadales* order*,* while females exhibited a greater abundance of *Lactobacillus* genus. These findings align with previous reports of sex-specific diversity in oral biofilms of periodontitis patients, including our prior studies in initial (Zhao et al., 2021) and severe periodontitis (Z et al., 2021) and a meta-analysis (Del Pinto et al., 2024), potentially explaining the higher male prevalence of periodontitis (Vale et al., 2018). However, the specific taxa identified across these studies differ from those in our work, suggesting that age and disease stage may modulate these patterns. A recent study reported that dental fluorosis may contribute to periodontitis and observed increased *Pseudomonadales* order abundance in the oral microbiota of affected patients (Liu et al., 2023). This supports the possibility that *Pseudomonadales* may be involved in periodontitis progression in males. The enrichment of *Lactobacillus* in females belongs to beneficial genera, as this genus has been associated with reduced intestinal inflammation through lactate production and TLR signaling modulation (Sun et al., 2026). In addition, *Lactobacillus rhamnosus* and *Lactobacillus acidophilus* supplementation have been shown to reduce the levels of pro-inflammatory cytokines and concurrently enhancing the expression of anti-inflammatory cytokines, thereby significantly affecting the severity of apical periodontitis in rats (Cosme-Silva et al., 2019). *Lactobacillus rhamnosus GG* administration effectively inhibited alveolar bone loss and gingival inflammation in periodontitis models (Gatej et al., 2018). However, the role of *Lactobacillus* in oral health is species-dependent. Some studies suggest that *Lactobacillus* is a cariogenic bacterium with the ability to produce and tolerate acids, and their detection rates are relatively high in deep carious lesions (Jiang et al., 2018). The lack of species-level resolution in our 16S-based approach is a limitation, and future studies employing metagenomic sequencing are needed to clarify which species of *Pseudomonadales* order and *Lactobacillus* genus underlie the observed sex-specific enrichment and clarify their functional roles in periodontitis.

KEGG pathway analysis revealed a trend of predicted upregulated cardiovascular disease pathways in males. This aligns with the established link between periodontitis and cardiovascular disease, and with evidence that men have higher risks of both conditions (Del Pinto et al., 2024; Yamazaki and Kamada, 2024; Nour et al., 2023). These convergent findings suggest that shared mechanisms may underlie male susceptibility to both diseases, warranting further investigation. Conversely, females showed a trend of enriched Immune system pathways and Metabolic related pathways (Amino acid metabolism, Carbohydrate metabolism, Lipid metabolism) at level 2, which may be associated with periodontal protection. Notably, a meta-analysis also reported sexually dimorphic immune activation during periodontitis, which is consistent with our findings (Del Pinto et al., 2024). The immune system has been established as playing a critically important role in the pathogenesis of periodontitis (Mo et al., 2024). Periodontitis is a disease caused by the imbalanced interaction between the local microbiome and the host immune response. Immunosenescence has been implicated in both periodontal dysbiosis and disease progression (Liu et al., 2022). Females showed a trend of enrichment of Immune system pathways, suggesting that sex-specific immune responses may be associated with the observed microbial differences. Interestingly, functional prediction at level 3 revealed a significant enrichment of the Hematopoietic cell lineage pathway in the females. This pathway is traditionally linked to immune activation, and recent immunological consensus indicates that inflammation resolution is an active process dependent on hematopoietic differentiation. Its upregulation may reflect a shift toward immune homeostasis and tissue repair. Favorable changes in the oral microbiome could modulate hematopoietic trajectories, promoting anti-inflammatory cell subsets (Sezaki et al., 2022; Zeng et al., 2023). These processes help control local inflammation and facilitate tissue healing. Enhanced Carbohydrate metabolism observed in females could help maintain oral homeostasis (Belstrøm et al., 2021). Amino acid metabolism modulation could suppress periodontal inflammation and alleviate bone loss (Yang et al., 2023). Notably, *Lactobacillus* in the gut microbiota can exert probiotic effects by modulating butyric acid levels, thereby maintaining intestinal barrier integrity and microbial balance (Chen et al., 2025). Additionally, altered lipid metabolism profiles in gingival tissues may serve as potential biomarkers for periodontitis (Zhou et al., 2022). Our findings suggest that the sex differences in subgingival microbiome composition may involve immune and metabolic regulatory mechanisms.

Oral microbiota dysbiosis is a key driver of periodontitis and alveolar bone loss (Scannapieco and Dongari-Bagtzoglou, 2021). Persistent chronic periodontal inflammation disrupts the coupling between osteoclast and osteoblast activity, ultimately leading to alveolar bone destruction (Hathaway-Schrader and Novince, 2000). Emerging evidence suggests that probiotic interventions can effectively control periodontitis and alveolar bone loss (Shimabukuro et al., 2021; Lucateli et al., 2024; Cataruci et al., 2024; Moraes et al., 2020). These findings suggest that the potential relevance of the observed sex-specific microbial differences to alveolar bone loss warrants further investigation. These results suggest that sex-specific considerations in periodontal therapy may be valuable. Probiotic interventions targeting *Lactobacillus* (e.g., specific strains) have shown potential in modulating oral health (Lin et al., 2022), but their application in sex-targeted strategies requires further investigation (Alves et al., 2025).

However, this exploratory cross-sectional study has a limited sample size and cannot establish causality; therefore, findings should be interpreted cautiously and require validation in larger, longitudinal studies with balanced groups. Unmeasured confounders—including menopausal status, dietary factors, oral hygiene practices, and medication use—should be addressed in future research, though our study population from a medical examination center likely represents a health-conscious cohort with relatively good oral hygiene. The use of 97% OTU clustering, while standard, offers lower resolution than ASV approaches, which should be considered in future studies. The functional pathways represent predictions based on 16S rRNA data, which require additional metagenomic or metatranscriptomic analysis and experiments for validation. Due to the limited sample size, correlation analyses with clinical parameters were not performed. The mechanistic uncertainties highlight the need for larger cohorts to validate our findings and elucidate the roles of *Lactobacillus* in periodontitis, particularly their interactions with immune and metabolic pathways. Their potential relevance to alveolar bone loss also warrants further investigation. Additionally, the potential therapeutic implications of sex-specific microbiome differences warrant further exploration.

## 5 Conclusion

This exploratory study observed sex-specific variations in subgingival microbiome of elderly patients with moderate periodontitis. Notably, females exhibited a distinct microbial profile characterized by an enriched abundance of *Lactobacillus*, which may be associated with predicted Immune system and Metabolic pathways. These findings suggest that sex-specific microbiome differences may be a relevant biological variable in future periodontitis research, and their potential link to alveolar bone loss and implications for periodontal therapy warrant further investigation.

## Data Availability

The original contributions presented in the study are publicly available. The raw 16S rRNA sequencing data presented in this study are deposited in the NCBI repository under accession number PRJNA763726 and PRJNA1197762. The customized R scripts and analytical pipelines used for data processing, supported by OE Biotech, are available from the corresponding author upon reasonable request.

## Supplementary material

SUPPLEMENTARY FIGURE S1 OTU level barplot of each participant at all levels of classification.

SUPPLEMENTARY TABLE S1 α-diversity summary.

SUPPLEMENTARY TABLE S2 Sequencing quality control metrics.

SUPPLEMENTARY TABLE S3 Full taxonomic composition.

## References

[B1] Abu Fanas S. Brigi C. Varma S. R. Desai V. Senok A. D'Souza J. (2021). The prevalence of novel periodontal pathogens and bacterial complexes in stage II generalized periodontitis based on 16S rRNA next generation sequencing. J. Appl. Oral Sci. 29, e20200787. 10.1590/1678-7757-2020-0787 34008792 PMC8128322

[B2] Abusleme L. Hoare A. Hong B. Y. Diaz P. I. (2000). Microbial signatures of health, gingivitis, and periodontitis. Periodontol 86 (1), 57–78. 10.1111/prd.12362 33690899

[B3] Alghamdi A. S. Almarghlani A. A. (2019). Periodontal pathogenic bacteria among high school children in Saudi Arabia. Ann. Saudi Med. 39 (4), 244–250. 10.5144/0256-4947.2019.244 31381369 PMC6838648

[B4] Alves T. Swanson K. V. Girnary M. S. Freire M. Moss K. Divaris K. (2025). Inflammasome targeting for periodontitis prevention is sex dependent. Proc. Natl. Acad. Sci. U. S. A. 122 (44), e2507092122. 10.1073/pnas.2507092122 41144672 PMC12595481

[B5] Arredondo A. Àlvarez G. Isabal S. Teughels W. Laleman I. Contreras M. J. (2023). Comparative 16S rRNA gene sequencing study of subgingival microbiota of healthy subjects and patients with periodontitis from four different countries. J. Clin. Periodontol. 50 (9), 1176–1187. 10.1111/jcpe.13827 37246304

[B6] Belstrøm D. Constancias F. Drautz-Moses D. I. Schuster S. C. Veleba M. Mahé F. (2021). Periodontitis associates with species-specific gene expression of the oral microbiota. NPJ Biofilms Microbiomes 7 (1), 76. 10.1038/s41522-021-00247-y 34556654 PMC8460658

[B7] Cataruci A. C. S. Kawamoto D. Shimabukuro N. Ishikawa K. H. Ando-Suguimoto E. S. Ribeiro R. A. (2024). Oral administration of Lactobacillus acidophilus LA5 prevents alveolar bone loss and alters oral and gut microbiomes in a murine periodontitis experimental model. Microorganisms 12 (6), 1057. 10.3390/microorganisms12061057 38930439 PMC11205731

[B8] Chen H. Zhao R. Xiao Z. Li Y. Yang J. Jiang S. (2025). Investigating the effects of Yunnan lufeng aromatic vinegar intervention on intestinal microbiota, SCFAs, and metabolites in mice using multi-omics techniques. Foods 14 (21), 3747. 10.3390/foods14213747 41227716 PMC12609717

[B9] Cornejo Ulloa P. van der Veen M. H. Brandt B. W. Buijs M. J. Krom B. P. (2023). The effect of sex steroid hormones on the ecology of *in vitro* oral biofilms. Biofilm 6, 100139. 10.1016/j.bioflm.2023.100139 37621393 PMC10447177

[B10] Cosme-Silva L. Dal-Fabbro R. Cintra L. T. A. Dos Santos V. R. Duque C. Ervolino E. (2019). Systemic administration of probiotics reduces the severity of apical periodontitis. Int. Endod. J. 52 (12), 1738–1749. 10.1111/iej.13192 31356689

[B11] DeClercq V. Nearing J. T. Langille M. G. I. (2021). Investigation of the impact of commonly used medications on the oral microbiome of individuals living without major chronic conditions. PLoS One 16 (12), e0261032. 10.1371/journal.pone.0261032 34882708 PMC8659300

[B12] Del Pinto R. Ferri C. Giannoni M. Cominelli F. Pizarro T. T. Pietropaoli D. (2024). Meta-analysis of oral microbiome reveals sex-based diversity in biofilms during periodontitis. JCI Insight 9 (17), e171311. 10.1172/jci.insight.171311 39253976 PMC11385077

[B13] Gatej S. M. Marino V. Bright R. Fitzsimmons T. R. Gully N. Zilm P. (2018). Probiotic Lactobacillus rhamnosus GG prevents alveolar bone loss in a mouse model of experimental periodontitis. J. Clin. Periodontol. 45 (2), 204–212. 10.1111/jcpe.12838 29121411

[B14] Gürsoy U. K. Oikonomou I. Yilmaz M. Gürsoy M. (2025). Advances in periodontal healing biomarkers. Adv. Clin. Chem. 125, 143–167. 10.1016/bs.acc.2024.11.007 39988405

[B15] Hajishengallis G. Chavakis T. Lambris J. D. (2000). Current understanding of periodontal disease pathogenesis and targets for host-modulation therapy. Periodontol 84 (1), 14–34. 10.1111/prd.12331 32844416 PMC7457922

[B16] Hathaway-Schrader J. D. Novince C. M. (2000). Maintaining homeostatic control of periodontal bone tissue. Periodontol 86 (1), 157–187. 10.1111/prd.12368 33690918 PMC8294452

[B17] Hirtz C. O'Flynn R. Voisin P. M. Deville de Périère D. Lehmann S. Guedes S. (2021). The potential impact of salivary peptides in periodontitis. Crit. Rev. Clin. Lab. Sci. 58 (7), 479–492. 10.1080/10408363.2021.1907298 33849374

[B18] Iniesta M. Chamorro C. Ambrosio N. Marín M. J. Sanz M. Herrera D. (2023). Subgingival microbiome in periodontal health, gingivitis and different stages of periodontitis. J. Clin. Periodontol. 50 (7), 905–920. 10.1111/jcpe.13793 36792073

[B19] Jaillon S. Berthenet K. Garlanda C. (2019). Sexual dimorphism in innate immunity. Clin. Rev. Allergy Immunol. 56 (3), 308–321. 10.1007/s12016-017-8648-x 28963611

[B20] Jia X. Yang R. Li J. Zhao L. Zhou X. Xu X. (2021). Gut-bone axis: a non-negligible contributor to periodontitis. Front. Cell Infect. Microbiol. 11, 752708. 10.3389/fcimb.2021.752708 34869062 PMC8637199

[B21] Jiang Q. Liu J. Chen L. Gan N. Yang D. (2018). The oral microbiome in the elderly with dental caries and health. Front. Cell Infect. Microbiol. 8, 442. 10.3389/fcimb.2018.00442 30662876 PMC6328972

[B22] Kumar M. Prakash S. Lorenzo J. M. Chandran D. Dhumal S. Dey A. (2022). Apitherapy and periodontal disease: insights into *in vitro*, *in vivo*, and clinical studies. Antioxidants (Basel) 11 (5), 823. 10.3390/antiox11050823 35624686 PMC9137511

[B23] Lin C. W. Chen Y. T. Ho H. H. Hsieh P. S. Kuo Y. W. Lin J. H. (2022). Lozenges with probiotic strains enhance oral immune response and health. Oral Dis. 28 (6), 1723–1732. 10.1111/odi.13854 33749084

[B24] Liu J. Dan R. Zhou X. Xiang J. Wang J. Liu J. (2022). Immune senescence and periodontitis: from mechanism to therapy. J. Leukoc. Biol. 112 (5), 1025–1040. 10.1002/JLB.3MR0822-645RR 36218054

[B25] Liu S. Song Q. Zhang C. Li M. Li Z. Liu Y. (2023). Saliva microbiome alterations in dental fluorosis population. J. Oral Microbiol. 15 (1), 2180927. 10.1080/20002297.2023.2180927 36844898 PMC9946311

[B26] Lucateli R. L. Silva P. H. F. Salvador S. L. Ervolino E. Furlaneto F. A. C. Marciano M. A. (2024). Probiotics enhance alveolar bone microarchitecture, intestinal morphology and estradiol levels in osteoporotic animals. J. Periodontal Res. 59 (4), 758–770. 10.1111/jre.13256 38699835

[B27] Mo K. Wang Y. Lu C. Li Z. (2024). Insight into the role of macrophages in periodontitis restoration and development. Virulence 15 (1), 2427234. 10.1080/21505594.2024.2427234 39535076 PMC11572313

[B28] Moraes R. M. Lescura C. M. Milhan N. V. M. Ribeiro J. L. Silva F. A. Anbinder A. L. (2020). Live and heat-killed Lactobacillus reuteri reduce alveolar bone loss on induced periodontitis in rats. Arch. Oral Biol. 119, 104894. 10.1016/j.archoralbio.2020.104894 32950917

[B29] Nour J. Bonacina F. Norata G. D. (2023). Gonadal sex vs genetic sex in experimental atherosclerosis. Atherosclerosis 384, 117277. 10.1016/j.atherosclerosis.2023.117277 37775425

[B30] Orishko A. Imber J. C. Roccuzzo A. Stähli A. Salvi G. E. (2000). Tooth- and implant-related prognostic factors in treatment planning. Periodontol 95 (1), 102–128. 10.1111/prd.12597 39234949

[B31] Scannapieco F. A. Dongari-Bagtzoglou A. (2021). Dysbiosis revisited: understanding the role of the oral microbiome in the pathogenesis of gingivitis and periodontitis: a critical assessment. J. Periodontol. 92 (8), 1071–1078. 10.1002/JPER.21-0120 33902163 PMC8380683

[B32] Segata N. Izard J. Waldron L. Gevers D. Miropolsky L. Garrett W. S. (2011). Metagenomic biomarker discovery and explanation. Genome Biol. 12 (6), R60. 10.1186/gb-2011-12-6-r60 21702898 PMC3218848

[B33] Sezaki M. Hayashi Y. Nakato G. Wang Y. Nakata S. Biswas S. (2022). Hematopoietic stem and progenitor cells integrate microbial signals to promote post-inflammation gut tissue repair. EMBO J. 41 (22), e110712. 10.15252/embj.2022110712 36254590 PMC9670188

[B34] Shimabukuro N. Cataruci A. C. S. Ishikawa K. H. de Oliveira B. E. Kawamoto D. Ando-Suguimoto E. S. (2021). Bifidobacterium strains present distinct effects on the control of alveolar bone loss in a periodontitis experimental model. Front. Pharmacol. 12, 713595. 10.3389/fphar.2021.713595 34630089 PMC8497694

[B35] Sun J. Shi J. Zhang J. Sun X. Du S. Wang X. (2026). Traditional Mongolian medicine Batri-7 exhibits chemopreventive activity in colitis-associated colorectal cancer through microbiota modulation and NLRP3 inflammasome targeting. J. Ethnopharmacol. 355 (Pt B), 120696. 10.1016/j.jep.2025.120696 41047048

[B36] Tonetti M. S. Greenwell H. Kornman K. S. (2018). Staging and grading of periodontitis: framework and proposal of a new classification and case definition. J. Periodontology 89 (Suppl. 1), S159–s172. 10.1002/JPER.18-0006 29926952

[B37] Topcuoglu N. Kulekci G. (2015). 16S rRNA based microarray analysis of ten periodontal bacteria in patients with different forms of periodontitis. Anaerobe 35 (Pt A), 35–40. 10.1016/j.anaerobe.2015.01.011 25638399

[B38] Valerio M. S. Kirkwood K. L. (2018). Sexual dimorphism in immunity to oral bacterial diseases: intersection of neutrophil and osteoclast pathobiology. J. Dent. Res. 97 (13), 1416–1423. 10.1177/0022034518798825 30205018 PMC6262266

[B39] Van der Velden U. (2000). What exactly distinguishes aggressive from chronic periodontitis: is it mainly a difference in the degree of bacterial invasiveness? Periodontol 75 (1), 24–44. 10.1111/prd.12202 28758297

[B40] Wei Y. Deng Y. Ma S. Ran M. Jia Y. Meng J. (2021). Local drug delivery systems as therapeutic strategies against periodontitis: a systematic review. J. Control Release 333, 269–282. 10.1016/j.jconrel.2021.03.041 33798664

[B41] Yamazaki K. Kamada N. (2024). Exploring the oral-gut linkage: interrelationship between oral and systemic diseases. Mucosal Immunol. 17 (1), 147–153. 10.1016/j.mucimm.2023.11.006 38007003 PMC11222583

[B42] Yang H. Han N. Luo Z. Xu J. Guo L. Liu Y. (2023). D-mannose alleviated alveolar bone loss in mice with experimental periodontitis *via* regulating the anti-inflammatory effect of amino acids. J. Periodontol. 94 (4), 542–553. 10.1002/JPER.22-0294 36031720

[B43] Yang S. Zhu Y. Ji C. Zhu H. Lao A. Zhao R. (2024). A five-in-one novel MOF-Modified injectable hydrogel with thermo-sensitive and adhesive properties for promoting alveolar bone repair in periodontitis: antibacterial, hemostasis, immune reprogramming, pro-osteo-/angiogenesis and recruitment. Bioact. Materials 41, 239–256. 10.1016/j.bioactmat.2024.07.016 39149594 PMC11324614

[B44] Yue Z. Li C. Yan F. Guan S. Fan Y. Chen X. (2025). The oral microbiome in aging: a window into health and longevity. J. Oral Microbiol. 17 (1), 2589648. 10.1080/20002297.2025.2589648 41341205 PMC12671430

[B45] Zhao Y. Q. Zhou Y. H. Zhao J. Feng Y. Gao Z. R. Ye Q. (2021). Sex variations in the oral microbiomes of youths with severe periodontitis. J. Immunology Research 2021, 8124593. 10.1155/2021/8124593 34722781 PMC8550847

[B46] Zeng X. Li X. Li X. Wei C. Shi C. Hu K. (2023). Fecal microbiota transplantation from young mice rejuvenates aged hematopoietic stem cells by suppressing inflammation. Blood 141 (14), 1691–1707. 10.1182/blood.2022017514 36638348 PMC10646769

[B47] Zhao J. Zhou Y. H. Zhao Y. Q. Feng Y. Yan F. Gao Z. R. (2021). Gender variations in the oral microbiomes of elderly patients with initial periodontitis. J. Immunology Research 2021, 7403042. 10.1155/2021/7403042 34859107 PMC8632398

[B48] Zhou M. Li C. Han X. Yu B. Yan X. Z. Zhang Y. (2022). Lipidomic analysis reveals altered lipid profiles of gingival tissues with periodontitis. J. Clin. Periodontol. 49 (11), 1192–1202. 10.1111/jcpe.13710 35924763

