# Supplementary figures and images for: Sex-specific variations in subgingival microbiome of elderly patients with moderate periodontitis: an exploratory study
Source: Front Genet. 2026 Apr 24;17:1791446. doi: 10.3389/fgene.2026.1791446 (PMC13152674; doi:10.3389/fgene.2026.1791446)

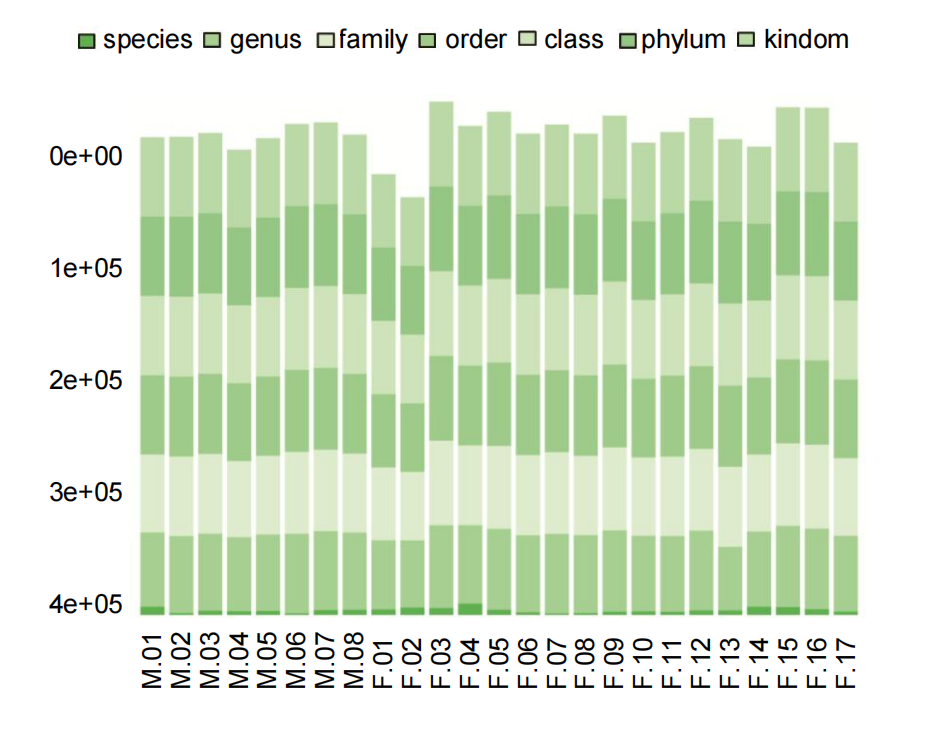

Supplement: Supplementary file 3 [file Image1.tif]
